# Exploiting Graphoelements and Convolutional Neural Networks with Long Short Term Memory for Classification of the Human Electroencephalogram

**DOI:** 10.1038/s41598-019-47854-6

**Published:** 2019-08-06

**Authors:** P. Nejedly, V. Kremen, V. Sladky, J. Cimbalnik, P. Klimes, F. Plesinger, I. Viscor, M. Pail, J. Halamek, B. H. Brinkmann, M. Brazdil, P. Jurak, G. Worrell

**Affiliations:** 1 Mayo Systems Electrophysiology Laboratory, Department of Neurology, Mayo Clinic, Rochester, MN 55905 USA; 2 Department of Neurology, St. Anne’s University Hospital, Brno, Czech Republic; 3 The Czech Academy of Sciences, Institute of Scientific Instruments, Brno, Czech Republic; 4 Department of Physiology and Biomedical Engineering, Mayo Clinic, Rochester, MN 55905 USA; 5 Czech Institute of Informatics, Robotics, and Cybernetics, Czech Technical University in Prague, Prague, Czech Republic; 6 International Clinical Research Center, St. Anne’s University Hospital Brno, Brno, Czech Republic

**Keywords:** Machine learning, Neurology

## Abstract

The electroencephalogram (EEG) is a cornerstone of neurophysiological research and clinical neurology. Historically, the classification of EEG as showing normal physiological or abnormal pathological activity has been performed by expert visual review. The potential value of unbiased, automated EEG classification has long been recognized, and in recent years the application of machine learning methods has received significant attention. A variety of solutions using convolutional neural networks (CNN) for EEG classification have emerged with impressive results. However, interpretation of CNN results and their connection with underlying basic electrophysiology has been unclear. This paper proposes a CNN architecture, which enables interpretation of intracranial EEG (iEEG) transients driving classification of brain activity as normal, pathological or artifactual. The goal is accomplished using CNN with long short-term memory (LSTM). We show that the method allows the visualization of iEEG graphoelements with the highest contribution to the final classification result using a classification heatmap and thus enables review of the raw iEEG data and interpret the decision of the model by electrophysiology means.

## Introduction

The interpretation of human brain electrical activity is fundamentally important as a cornerstone of neuroscience research and medicine. The electrical activity of the brain, measured by scalp or intracranial electroencephalography (EEG, iEEG), is a time series of voltage changes that can be measured from the scalp or on the brain surface. The EEG has long been a focus of both scientific and clinical brain research^1,2^. Epilepsy is a disorder characterized by unprovoked, recurrent seizures and has many underlying causes, but is unified by the common clinical expression of spontaneous seizures and the associated pathological electrical activity recorded with scalp EEG and iEEG. In addition, the EEG often shows sporadic, transient, abnormalities during periods without seizures and apparent normal brain function. Not long after the discovery of the human EEG, Hans Berger also reported that epileptic seizures were associated with an abnormal EEG and that even between the seizures (interictal) there were transient epileptiform voltage abnormalities, called spikes or interictal epileptiform discharges, that are not seen in subjects without epilepsy^3^. The significance of the interictal epileptiform spikes, electrographic seizures, and other abnormal transient oscillatory activity remain active areas of epilepsy research and clinical practice^4^. Visual EEG interpretation is subjective, and the inter-rater reproducibility is a well-recognized challenge^5–10^ The challenge of visually identifying EEG abnormalities and artifacts in long-term, multi-channel data has motivated the development of automated quantitative EEG^4^. There are, however, fundamental challenges with the automated classification of EEG as abnormal, artifacts, or normal.

Convolutional Neural Networks (CNNs) have demonstrated usefulness in a wide variety of industrial and scientific fields, including image recognition^11^, speech recognition^12^, biological signal processing and reinforcement learning^13^. CNNs have proven to be superior to traditional signal processing techniques in ECG and polysomnography classification during several challenges^14,15^ and have been used in variety of EEG processing tasks^16^, for example iEEG noise detection^17^, epileptic seizure detection^18^ and seizure prediction^19^.

In previous work^17^ we developed a CNN method for differentiation of iEEG signals between artifact (machine acquisition artifacts and muscle artifacts), physiological activity and pathological epileptiform activity. The technique allowed large-scale data processing of wide bandwidth iEEG recordings (0.01–900 Hz; sampling frequency 5 kHz) with hundreds of channels spanning multiple days, with datasets reaching hundreds of gigabytes. We also demonstrated a transfer learning method for easy retraining and adaptation to new datasets from different acquisition systems and institutions.

While the power of CNNs for classification is well recognized, a limitation has been the inability to make a direct connection between CNN results and the iEEG signals that have well established spectral features and clinical correlates^20^. Here we address the lack of interpretability using a Long short-term memory (LSTM) neural network. LSTM are a sub-type of recurrent neural networks (RNN) commonly used for time series classification and prediction^21^. The main advantage of RNN architecture is the processing of input features in sequential temporal order, and the ability to process sequences of different lengths. However, training of RNNs is computationally expensive and can be numerically unstable (exploding and vanishing gradients). LSTMs are designed to help resolve the numerical instability during training^22^.

Here we extended our previously described approach^17^ and developed and validated a Convolutional-LSTM neural network architecture, which enables partial interpretation of the CNN results using classification heatmaps. This allows for a visual inspection and correlation of the raw iEEG with the data segments driving the classification of the network, and extends our ability to interpret the CNN results, which was not possible with the previous method. Furthermore, we show that compared to the previous approach, the method reaches comparable classification results in out of institution testing on a different EEG acquisition system, at the expense of increased computational time for model training.

## Results

**Figure 1:**
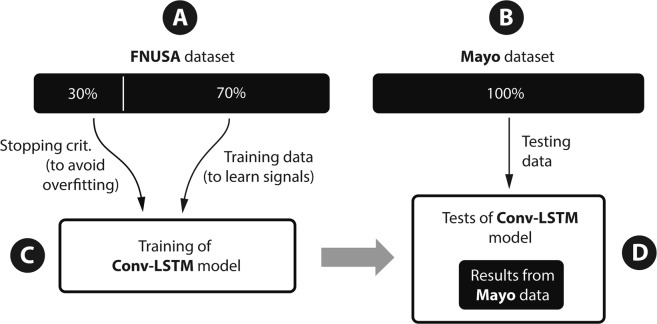
Training scheme for out of institution testing using different acquisition system. The model was trained and validated on data from St Anne’s University Hospital and tested on an out of sample and out of institution Mayo Clinic dataset. Recordings from both datasets were obtained under different conditions that included different acquisition systems, behavioral states (wake versus sleep), and patients.

The model was trained on the St Anne’s University Hospital dataset and tested on the Mayo Clinic dataset (out of institution testing), acquired with a different acquisition system in different recording conditions (Fig. 1). Proposed datasets are described in methods. The results from the CNN and the new Convolutional LSTM model are compared in Table 1. The results of LSTM network suggest similar performance (average F1-score) to the previously described CNN method with advantage that the LSTM enables visualization of the decision process of the neural network.

**Table 1:** Comparison of proposed Convolutional LSTM method with the previous method. Classification label is selected as group with highest probability i.e. argmax function of softmax output. Table describes results in terms of sensitivity (SEN), positive prediction value (PPV) and F1 score.

	CNN method from Nejedly, Cimbalnik, *et al*. 2018			Convolutional LSTM		
Classification category	F1	PPV	SEN	F1	PPV	SEN
Physiological	**0.90**	0.93	0.87	**0.86**	0.85	0.87
Pathological	**0.64**	0.57	0.74	**0.73**	0.66	0.82
Artifacts	**0.89**	0.88	0.91	**0.80**	0.85	0.76
Average	**0.81**	0.79	0.84	**0.80**	0.78	0.82

**Figure 2:**
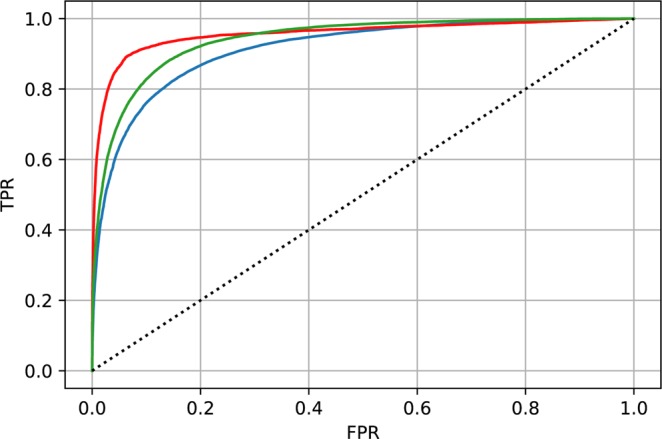
Receiver operating characteristic (ROC) for each classification group (blue - physiological, red - pathological, green - noise).

**Figure 3:**
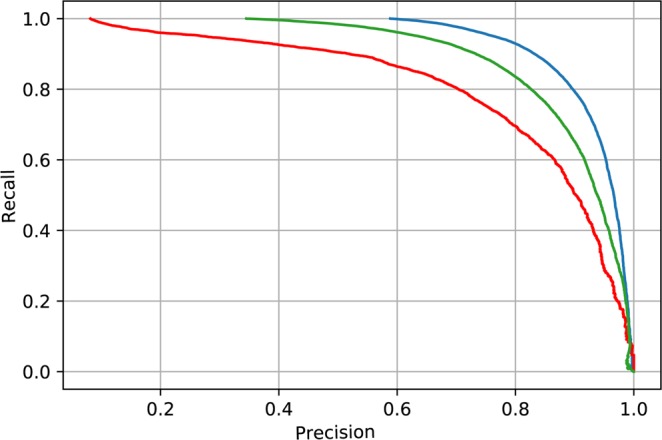
Precision recall curve (PRC) for each classification group (blue - physiological, red - pathological, green - noise).

Moreover, we report Received-Operating Curves (ROC, Fig. 2) and precision recall curve (PRC, Fig. 3) for each classification category. This allows users to set custom probability thresholds for each class independently and permits tailoring approaches to the particular applications, for example hypersensitive detector followed by expert review (expert-in-the-loop scenario), which will likely be the best clinical approach. The area under the ROC is 0.91, 0.95, 0.94 and area under the PRC is 0.93, 0.81, 0.89 for physiological, pathological and noise classes, respectively. We would like to highlight the results in ROC and PRC in this given unbalanced data set problem. For example, classification of pathological class in ROC space shows best results (taking AUC), however PRC space indicates lowest score (AUC). This phenomenon occurs as a consequence of the unbalanced dataset and demonstrates the value of reporting PRC along with ROC. Since LSTM is initialized with random values, we have evaluated 30 random testing runs for each example in testing set. The random initialization of LSTM hidden state has a negligible effect for classification performance, standard deviation for each class in AUPRC space is lower than 0.001.

**Figure 4:**
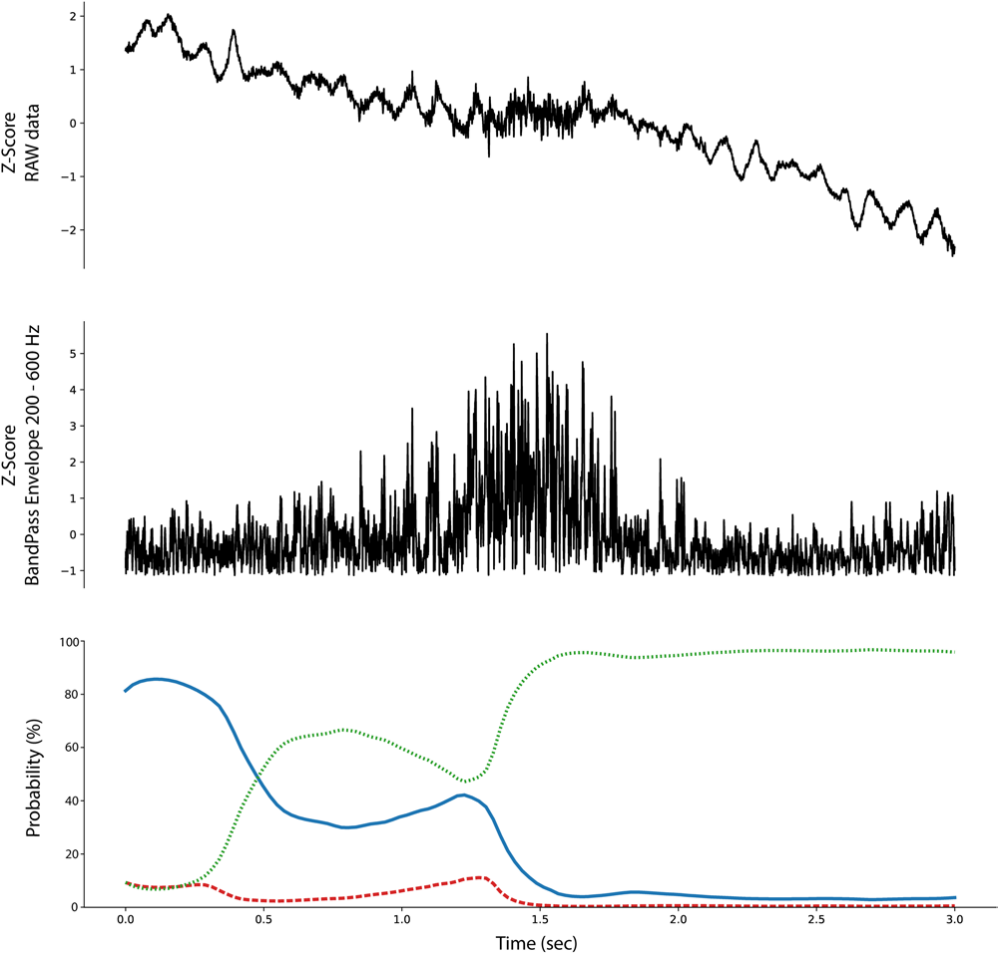
Raw iEEG data from testing dataset (top) contaminated with high-frequency noise caused by muscle artifact. The artifact in the data are created by the patient chewing. The center graph shows the raw-data signal bandpass filtered envelope (200–600 Hz) to highlight the high-frequency content in the data^23^. The bottom graph shows a probability heatmaps (green dotted line shows noise, blue full line shows physiology, and red dashed line shows pathology).

**Figure 5:**
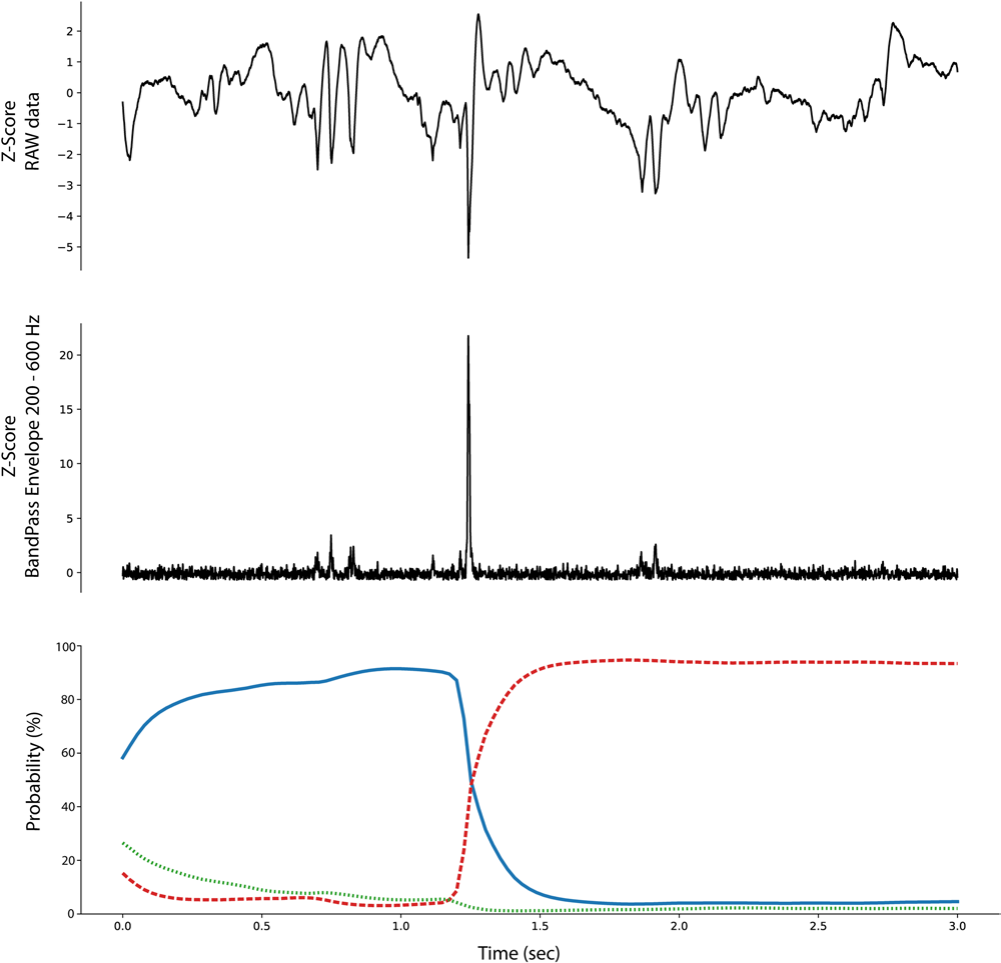
Raw iEEG data from testing dataset (top) and the interictal epileptiform spike at 1.2 sec with a superimposed high-frequency oscillation riding on the epileptiform spike. The middle graph shows the bandpass filtered signal envelope to highlight high-frequency content^23^. Bottom graph shows a probability heatmap (green dotted line shows noise, blue full line shows physiology, and red dashed line shows pathology).

**Figure 6:**
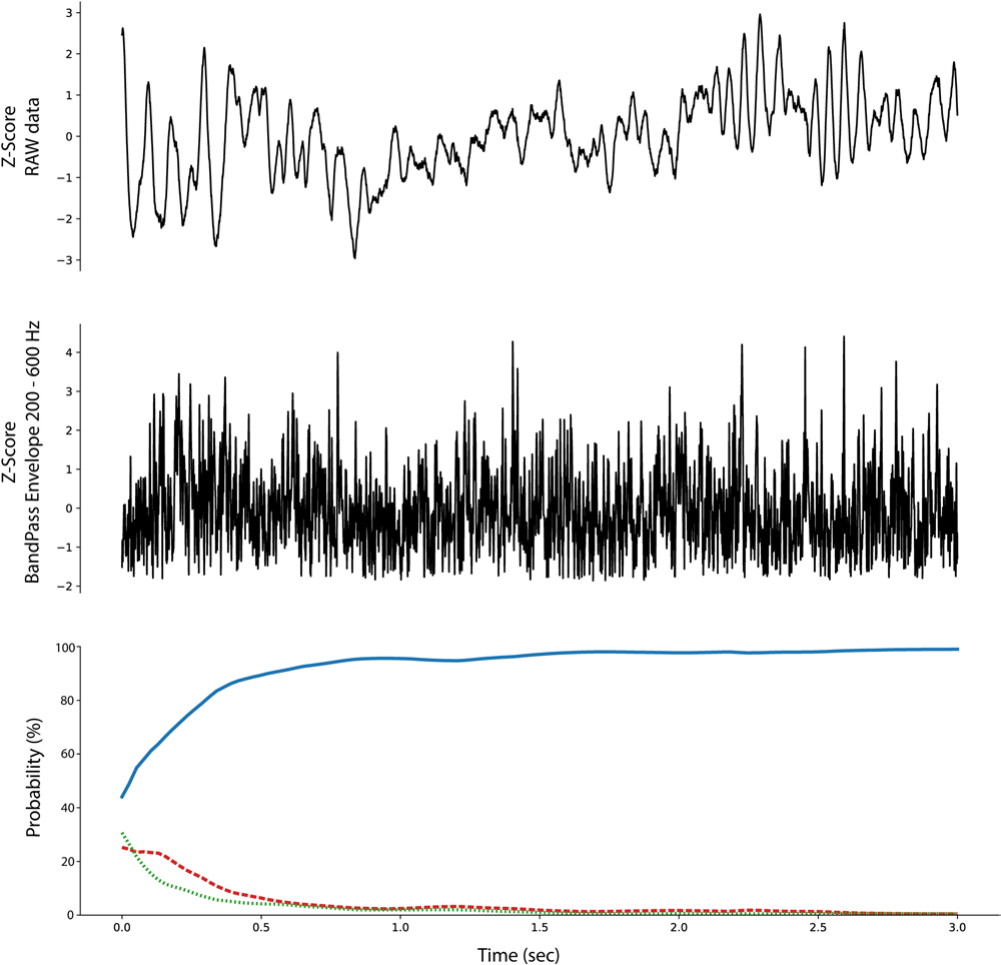
Example of raw iEEG data from testing dataset (top) without pathological graphoelements (spike or HFO) and dominant beta band power (awake), which is correctly classified as physiological activity. The middle graph of the bandpass filtered signal envelope showing lack of high-frequency activity with significant power (compared to Figs 4 and 5). Bottom graph shows a probability heatmap (green dotted line shows noise, blue full line shows physiology, and red dashed line shows pathology).

**Figure 7:**
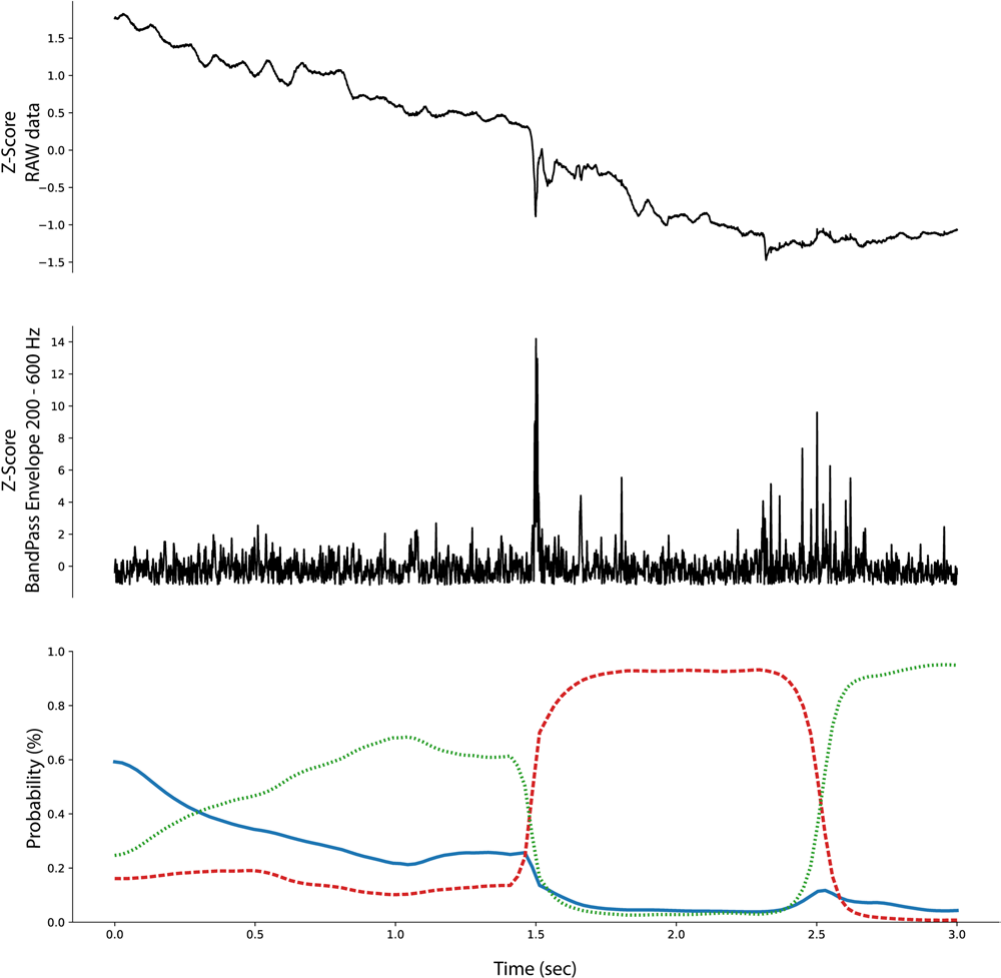
Example of raw iEEG data from testing dataset (top) with pathological graphoelements (spike or HFO) and high frequency noise activity. Results shows that the model switch to pathological state around 1.5 sec, while additional classification state switch occurs at 2.5 sec showing noise activity. The middle graph shows the bandpass filtered signal envelope high-frequency activity. Bottom graph shows a probability heatmap (green dotted line shows noise, blue full line shows physiology, and red dashed line shows pathology).

The ability to backtrack, visualize, and interpret the LSTM decision are shown for representative examples of each iEEG class in Figs 4–7. The figures show the original raw data and bandpass envelope (as one feature of the data) in high frequency bandwidth 200–600 Hz, which is commonly used for detection of HFOs and other high frequency properties of the raw electrophysiological data^23^. Below we show the probability heatmap to demonstrate where the LSTM switches from one class to another. For example, using a classification heatmap is shown in Fig. 5, where the proposed model is used for classification of an iEEG segment containing a HFO riding on an epileptiform spike. The internal state of the model classifies segments as physiological until the epileptiform spike with the HFO is detected, subsequently LSTM changes classification state to the Pathological group, which is a true positive classification. In addition, we show behavior of the model on pure iEEG data with a dominant physiological beta activity (Fig. 6) and a segment contaminated with high frequency noise (Fig. 4). Figure 7 shows the model behavior when the iEEG segment contains graphoelements from two classes, i.e. pathological and noise. The classification heatmaps show the segments of iEEG driving the classification. The final classification and assignment of the segment to a category (physiological, pathological, noise) is made based on the final timestamp, for this reason the segment in Fig. 7 is categorized as noise. This classification is counted as false because this segment was an expert labeled pathological segment because of the pathological spike and HFO. This example, points out the advantage of the current approach compared to our previous approach^17^, where interpretation of the false decision was not possible. Visual inspection of the heatmap reveals that model correctly detected pathological part, however was later over-voted to the noise state. This drawback might be removed by multiclass training, which would require creation of a new gold standard dataset e.g. this example would be annotated as pathological and noise. This situation can also be handled by cascade classification using the heatmaps in the postprocessing steps.

## Discussion and Conclusion

Automated processing and data mining from iEEG recordings with hundreds of channels spanning multiple days is possible with CNNs, however interpretation of results and why the CNN decides a particular classification has been unclear. Lack of ability to backtrack the decision process of CNN, makes it less interesting for use in clinical practice. The ability to localize graphoelements that drive final classification could improve the automated review, classification, and interpretation of iEEG recordings by providing visualization for greater clarity. For example, this might be used for supervised adaptive retraining in expert-in-the-loop scenario based on expert’s review of false positive or false negative classifications.

The proposed method based on convolutional LSTM neural networks has the ability to identify graphoelements with the highest contribution to the final classifier decision. Results are displayed by classification probability heatmaps i.e. time-series of probabilities for each classification class, which represents the internal state of LSTM model at a given time that can be linked to the raw data. Abrupt changes in heat-maps show graphoelements that drive the model to change internal classification state to a different class (Figs 4–6). The proposed model demonstrates this ability during classification of iEEG segments into three groups (physiological, pathological, and artifacts).

In conclusion, the current paper introduces a Convolutional-LSTM neural network for classification and localization of iEEG events (physiological, pathological, and artifactual data) driving the classification by using classification heatmaps. The functionality of the proposed model was demonstrated on datasets with noise, physiological and pathological iEEG activity from St Anne’s University Hospital (Czech Republic) and Mayo Clinic (USA). We compared the new Convolutional-LSTM model with a current state of the art CNN using the same datasets (out of institution testing). The numerical results show comparable performance with the state-of-the-art methods, while the architecture of the Convolutional-LSTM and classification probability heat maps extends our understanding of the iEEG activity driving the classification with ability to visualize and interpret the result, using correlations of heatmaps with raw data. This latter feature was not possible with previous deep learning architectures^17^.

In current era of digital health, when big datasets are generated in many areas, such as in iEEG electrophysiology, the benefit of methods like we show here should be substantial for automated analysis and data-mining. Data collected in real-life, clinical, scenarios contain many artifacts and inconsistencies that make analysis difficult and might bias results. This is one of the primary motivations for the approach presented in this article. A common limitation of many model is, of course, that the training is specific to the acquisition system and institution where the iEEG data are collected. Here we present a model that generalized across institutions and different acquisition systems. The model can be used as an initial step in analyses to localize artifacts and pathological activity in other EEG applications, such as scalp EEG electrophysiology (sleep medicine, seizure onset zone localization, etc.). To do so, the initial model needs to be retrained by the transfer learning approach into to the target data domain.

## Methods

### Datasets

For the purpose of the study, we have used two iEEG datasets with 5 kHz sampling rates (St Anne’s University Hospital iEEG dataset and Mayo Clinic iEEG dataset) published in our previous study^17^, where a comprehensive description of the dataset is provided.

The St Anne’s dataset was created from 11 patients with epilepsy undergoing epilepsy pre-surgical invasive EEG monitoring, with recording conducted during awake interictal resting state^23^ using a custom acquisition system (M&I; Brainscope, Czech Republic). The Mayo Clinic dataset was obtained from 25 patients undergoing pre-surgical invasive monitoring with a different acquisition system (Neuralynx Inc., Bozeman MT). Recordings used for this study were obtained from each patient’s first over-night recording (1 AM–3 AM) following intracranial electrode implantation.

The datasets from each institution are described in Table 2. The iEEG recordings were manually reviewed and annotated in each individual channel separately using SignalPlant software^24,25^. The signal annotation and classification was conducted manually by three independent operators (persons not involved in data analysis) by a technique using power distribution matrices (PDM)l^23^. The PDM method utilize the Hilbert transform applied to bandpass filtered signal to highlight high frequency activity higher than certain threshold (derived from standard deviation of steady state physiological activity signal). Further, all detections were visually inspected in the raw data domain and classified into groups based on content of EEG graphoelements, physiological activity, pathological activity (interictal epileptiform spikes and HFOs), and artifacts (muscle artifacts, powerline distortion) and subsequently segmented into 3-second length segments (15,000 samples) using constant-length segmentation. The segments were classified into 3 mutually exclusive groups: physiological activity, pathological activity (interictal spikes and HFOs), and artifactual signals (machine and muscle artifacts).

**Table 2:** St Anne’s University Hospital and Mayo Clinic Datasets. The table shows the number of examples for each classification category.

Classification category	St. Anne’s University Hospital	Mayo Clinic
Physiological Activity	66581	44259
Pathological Activity	18184	6099
Artifacts	13777	25389
Total	98542	75747

### Data preprocessing and training scheme

The proposed method uses single channel inputs. Each channel is segmented to constant-segment-length with 15000 samples (3-seconds windows). This window-length was heuristically set to account for the fact that muscle artifacts may span over several seconds^17^. Each iEEG segment was converted into a spectrogram using a Short Time Fourier Transform (STFT, window 256 samples, overlap 128 samples, NFFT 1024 samples) and further normalized by calculating z-scores for each frequency band. Frequencies greater than 1 kHz were discarded. The STFT window of 256 samples was empirically chosen to identify fast transient events. The NFFT was selected as 1024 samples in order to set frequency bandwidth at approximately 5 Hz for each band. This yielded spectrograms with 200 frequency bands and T time steps, where T is the length of signal after application of STFT transform. Subsequently, the spectrograms were aggregated into 4-dimensional tensors in order for mini-batch processing^26^. Then, these tensors enter the convolutional LSTM neural network and each T time steps probabilities are calculated as a result of this step. Lastly, the results are interpolated from T time steps in STFT domain back to the original time-domain with 15000 samples resolution. This yields in detections that starts one STFT segment ahead of real event in the data (256 samples).

The proposed Convolutional-LSTM neural network requires extensive computational power in order to train the model. For this reason, the model was trained in the data-parallel scheme^27^ utilizing a dedicated deep-learning server with 8 GPUs (Lambda Blade, Lambda Labs Inc., San Francisco). Each GPU processed part of the mini-batch and computed gradients, then gradients were averaged and the model was updated with backpropagation through time. The Adam optimization technique^28^ was used for model training.

The model was tested in an out-of-institution scheme, which was introduced previously^17^. The out-of-institution training scheme provides a worst-case scenario model testing, where the classifier was trained on data from first institution, while testing the classifier on out-of-sample data from a different set of patients at a different institution using a different acquisition system. The data were acquired on two different acquisition systems (M&I; Brainscope, Czech Republic and Neuralynx Inc., Bozeman MT) for training and testing, respectively. Moreover, we have recorded the data for training in a different behavioral state (awake) compare to testing data where recording was performed mostly during sleep. This statistical model testing simulates model deployment in different environment, while performing the same task (detection of EEG graphoelements). The model was trained and validated on dataset from the first institution (St Anne’s University Hospital) and finally tested on a completely hidden dataset from the second institution (Mayo Clinic) providing unbiased statistics and eliminating any overtraining concerns (Fig. 1).

### Model architecture

**Figure 8:**
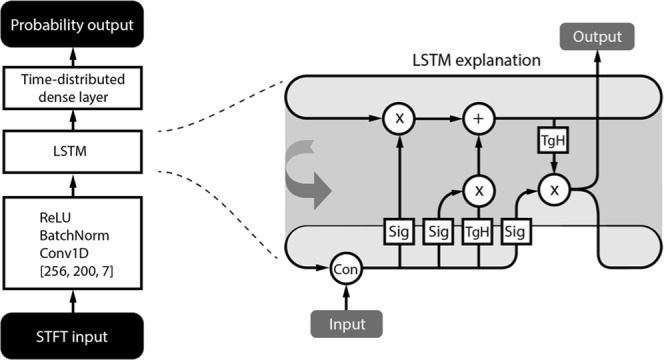
Architecture of the proposed neural network and detail description of the LSTM cell. TgH – Hyperbolic tangent activation function, Sig – Sigmoid activation function, Con – Vector concatenation, ReLU – rectified linear unit, STFT – Short Time Fourier Transform.

The proposed Convolutional-LSTM neural network (Fig. 8) was built in the PyTorch deep-learning framework^29^. The convolutional kernel for extraction of features from spectrograms consists of 256 filters with spatial dimensions 200 × 7, which processes all 200 frequency bands in groups of 7 time-steps. A batch normalization layer and rectified linear units (ReLU) were attached. Resulting latent feature space vectors are sequentially forwarded to unidirectional LSTM (256 input neurons and 128 output neurons) respecting the temporal arrangement. A, time-distributed fully connected layer with a softmax activation function is attached, providing classification probabilities for each spectrogram in mini-batch.

During the training process, the cross-entropy loss function was optimized with Adam optimizer. LSTM states are set as uniformly distributed random values during the model initialization, which helps with gradient propagation during the training phase, and helps prevent overtraining that might be caused by a static initialization^30^. The loss function was evaluated from the last sample of probability output and provided feedback to the network during (known as many to one training scheme). In general, it would be more efficient to train the model in many to many training scheme, however, for this type of training binary mask for each sample must be provided. This would require physician’s to precisely classify each time sample of iEEG segment into a classification category, which is not feasible. The dataset classifies each iEEG segment with just one classification category describing the whole segment. For example, if given segment is marked as pathological, this means that pathological event occurred somewhere in this segment, however the position is not precisely specified. The consequence of the many to one training scheme is that the model holds the state in order to correctly classify the last sample of given segment, which is enforced by minimizing the models’ loss function during the training process. For example, this behavior might be observed from Fig. 5, where the model holds the pathological state until the end of the given iEEG segment. If we had used a many to many training scheme, only the pathological part (spike and HFO) of the iEEG would be marked as pathological and the rest of signal would be marked as physiological.

During the inference phase the outputs for each time sample are reported (many to many scheme) which yields a probability time series (probability for each sample from a given segment), that we define as the classification heatmap (Bottom graphs in Figs 4–7). Abrupt changes in the classification heatmaps depicts graphoelements that significantly contribute to the final decision of the proposed model.

## Compliance with Ethical Standards

### Ethics statement

This study was carried out in accordance with the approval of the Mayo Clinic Institutional Review Board with written informed consent from all subjects. The protocol was approved by the Mayo Clinic Institutional Review Board and St. Anne’s University Hospital Research Ethics Committee and the Ethics Committee of Masaryk University. All subjects gave written informed consent in accordance with the Declaration of Helsinki.

## Data Availability

Overall size of the used data exceed several hundreds of gigabytes, cannot be publicly shared and requires special functions for decompression and reading (multiscale electrophysiology format (.mef) and d-file(.d)). However, data might be obtained upon request by contacting principal investigator of the projects. Mayo clinic data may be obtained through the web site of professor Gregory Worrell, M.D., Ph.D. (http://msel.mayo.edu) and St. Anne’s data might be obtained by contacting professor Milan Brazdil, M.D., Ph.D.
